# Hyponatremia and the Thyroid: Causality or Association?

**DOI:** 10.3390/jcm4010032

**Published:** 2014-12-26

**Authors:** Kevin M. Pantalone, Betul A. Hatipoglu

**Affiliations:** Department of Endocrinology, Cleveland Clinic, 9500 Euclid Avenue, Desk F-20, Cleveland, OH 44195, USA

**Keywords:** hyponatremia, thyroid, hypothyroidism, electrolytes

## Abstract

Thyroid disorders, particularly hypothyroidism, have historically been implicated in the development of serum hyponatremia. However, in more recent years, this paradigm has been challenged, and it has been suggested that the link between hypothyroidism and hyponatremia may merely be an association. This review will focus on the thyroid and its link with serum hyponatremia, and review the available literature on the topic.

## 1. Introduction

Alteration in thyroid function, particularly the development of hypothyroidism, has historically been linked to the development of serum hyponatremia. Although there is evidence to suggest that short-term uncomplicated hypothyroidism can result in a reduction in glomerular filtration rate (GFR) and a subsequent increase in serum creatinine [1,2], the data supporting the development of hyponatremia in this setting is limited and conflicting. Most of the literature supporting the development of hyponatremia secondary to hypothyroidism has been reported in the setting of myxedema coma, the most severe manifestation/presentation of untreated hypothyroidism, and in the inpatient setting. This review will focus on the thyroid and its association with serum hyponatremia, and review the available literature on the topic. 

## 2. Hypothyroidism and Hyponatremia

Hypothyroidism, along with adrenal insufficiency, syndrome of inappropriate antidiuretic hormone (SIADH), and primary polydipsia, has long been included in the differential diagnosis of euvolemic hyponatremia. Reports have suggested that the pathogenesis of “hypothyroid hyponatremia” is secondary to the inability to excrete a free water load, caused by both a decrease in the delivery of water to the distal nephron [3], as well as excess vasopressin secretion [4]. Derubertis *et al.* [3] suggested that the impaired water excretion in the setting of hypothyroidism is likely to be related to a reduction in renal perfusion (*i.e.*, a reduction in the GFR) secondary to the systemic effects of thyroid hormone deficiency on cardiac output and peripheral vascular resistance. It should be noted, however, that the patients under study in this report were those with myxedema coma, not routine cases of uncomplicated hypothyroidism. While elevations in serum creatinine have been reported to be common in patients with short-term uncomplicated hypothyroidism, concordant reductions in serum sodium has not been demonstrated consistently. Baajafer *et al.* [1] reported that elevation in serum creatinine in patients with short-term uncomplicated hypothyroidism was common, whereas hyponatremia was not. In contrast, Montenegro *et al.* [2] studied 41 patients with primary hypothyroidism before and after thyroid hormone replacement, and found that all patients had a decreased GFR (creatinine clearance and urea clearance in 24 h urine samples were used for estimating GFR), and 22 patients had an increased serum creatinine level. Hyponatremia was documented in 45% of the 22 patients with an elevated serum creatinine, but in only 21% of 10 patients with normal creatinine. All of these defects were reportedly corrected by treatment with thyroid hormone. Allon *et al.* [5] compared the tubular function in hypothyroid patients to patients with chronic kidney disease and normal subjects. The lithium clearance method and oral water loading were used to evaluate parameters of tubular sodium and water handling, respectively. The hypothyroid and the chronic kidney disease patients were selected to have similar reductions in glomerular filtration rate. They reported that the abnormalities in tubular sodium and water handling in hypothyroid patients was comparable to those present in other patients with a similar degree of renal insufficiency, suggesting that the tubular abnormalities in sodium and water excretion in hypothyroidism may be a consequence of the associated decrease in glomerular filtration rate.

The available literature suggests that only myxedema coma (a term often used inappropriately interchangeably with severe hypothyroidism in the literature) is capable of causing significant reductions in GFR that result in large enough disturbances in renal tubular function to cause clinically significant hyponatremia. Myxedema coma, characterized by chronic extreme thyroid-stimulating hormone (TSH) elevations, has repeatedly been linked to the development of hyponatremia [6,7,8], among many other complications. It is likely that the development of hyponatremia in the setting of myxedema coma is multifactorial, as multi-organ failure commonly occurs in this setting. However, data supporting the development of hyponatremia in more mild- to moderate-cases of hypothyroidism, as well as severe hypothyroidism (but without myxedema coma) are limited and conflicting; studies examining infants born with congenital hypothyroidism and adults with primary hypothyroidism have failed to establish a causal relationship [2,9,10,11,12,13]. The vast majority of evidence suggests that more moderate cases of hypothyroidism do not cause hyponatremia. Asami *et al*. [13] studied 32 neonates and infants with congenital hypothyroidism detected during routine screening, and found no difference in serum sodium levels when compared to 16 age-matched control neonates, 139.1 ± 1.5 mmol/L and 139.3 ± 1.3 mmol/L, respectively. Furthermore, no change in serum sodium values was observed in 25 of the hypothyroid neonates after 2 months of treatment with levothyroxine (LT4). More recently, Sun *et al.* [9] studied 10 severely hypothyroid patients undergoing an LT4 absorption test to evaluate for malabsorption *vs.* pseudomalabsorption and found that even in the setting of extreme elevations in serum TSH (median TSH 193 μU/mL, range: 104.2 to 515.6 μU/mL), hyponatremia was not observed. Larger scale studies have also arrived at a similar conclusion. Croal *et al.* [10] evaluated the prevalence of hyponatremia in a large group (*n* = 33,912) of unselected hypothyroid patients from a large city general hospital. The distribution of serum sodium values was similar in biochemically euthyroid (*N* = 30,375) and hypothyroid patients (*N* = 3537; of which *N* = 455 had a TSH >40 U/L). The proportion of euthyroid and hypothyroid patients with a serum sodium value below that of the normal reference range (135 mmol/L) was not significantly different between the euthyroid and hypothyroid subjects, 11.4% and 12.8%, respectively. Warner *et al.* [11] evaluated 999 newly diagnosed hypothyroid patients in an ambulatory setting, and reported that for every 10 mIU/L increase in serum TSH, only a 0.14 mEq/L drop in serum sodium was observed, hardly a decrement that is clinically significant.

Hyponatremia and hypothyroidism are very common in the general population, with either acute or chronic illness, and perhaps even more common in the hospitalized patient [14], thus their simultaneous occurrence is not surprising, and one should not conclude that their common occurrence establishes causality. The vast majority of cases of “hypothyroid hyponatremia” observed in clinical practice occur in the inpatient setting, where there are likely to be additional confounding factors and comorbidities that may be driving the development of the hyponatremia, rather than the hypothyroidism alone. In this setting, the possibility of glucocorticoid deficiency should be considered, as well as SIADH, a common confounding condition in the hospitalized setting that would need to be considered in the differential diagnosis of “hypothyroid hyponatremia”. Lastly, the majority of cases of “hypothyroid hyponatremia” are related to primary hypothyroidism. Clearly, in cases of secondary hypothyroidism, where a pituitary/hypothalamic etiology is suspected, the etiology of hyponatremia is likely to be multifactorial, and the presence of glucocorticoid deficiency must be considered [15].

## 3. Thyroid Cancer and Hyponatremia

There are ten reported cases in the literature of subjects that have developed hyponatremia while undergoing treatment for differentiated thyroid cancer [16,17,18,19,20]. All ten patients were placed on a low-iodine diet (the duration and severity of the dietary restrictions varied). In preparation of radioactive iodine ablation or treatment, eight of these patients underwent LT4 withdrawal and were thus hypothyroid; two were administered recombinant human thyrotropin. The two subjects who received recombinant human thyrotropin instead of undergoing LT4 withdrawal were both receiving hydrochlorothiazide; one of these two subjects was euthyroid; the other had a suppressed TSH at baseline. All subjects were >65 years of age. This data suggests that the development of hyponatremia in patients being prepared for radioactive iodine is not related to their thyroid status (hypothyroid, euthyroid, or hyperthyroid), rather their age, concomitant diuretic therapy, and the low iodine diet. The low-iodine diet is rather restrictive, resulting in a low overall solute intake, and it is likely to be the strong driver of the development of hyponatremia in these elderly patients, a population of patients in which renal impairment is not uncommon. Additionally, metastatic lesions themselves may also play a role in the development of hyponatremia in those subjects receiving radioactive iodine, as in the case reported by Shakir *et al.* [16], where pulmonary and/or brain mets were documented in 4 of the 5 subjects.

## 4. Conclusions

In conclusion, in more recent years, the paradigm that routine cases of hypothyroidism cause hyponatremia has been challenged [21,22]. Outside of severe cases of hypothyroidism, particularly those referred to as myxedema coma, hyponatremia of clinical relevance appears dubious. Although hyponatremia is still listed as an etiology of hyponatremia in the most recent expert panel recommendations [23], the authors do suggest that routine hypothyroidism as an etiology of hyponatremia is unlikely, and unless the hypothyroidism is severe (*i.e.*, symptoms and signs of myxedema or thyroid-stimulating hormone >50 mIU/mL), other causes of hyponatremia should be sought rather than ascribing the hyponatremia to hypothyroidism. 

## References

[1] Baajafer F.S., Hammami M.M., Mohamed G.E. (1999). Prevalence and severity of hyponatremia and hypercreatininemia in short-term uncomplicated hypothyroidism. J. Endocrinol. Investig..

[2] Montenegro J., Gonzalez O., Saracho R., Aguirre R., Gonzalez O., Martinez I. (1996). Changes in renal function in primary hypothyroidism. Am. J. Kidney Dis..

[3] Derubertis F.R., Michelis M.F., Bloom M.E., Mintz D.H., Field J.B., Davis B.B. (1971). Impaired water excretion in myxedema. Am. J. Med..

[4] Skowsky W.R., Kikuchi T.A. (1978). The role of vasopressin in the impaired water excretion of myxedema. Am. J. Med..

[5] Allon M., Harrow A., Pasque C.B., Rodriguez M. (1990). Renal sodium and water handling in hypothyroid patients: The role of renal insufficiency. J. Am. Soc. Nephrol..

[6] Curtis R.H. (1956). Hyponatremia in primary myxedema. Ann. Intern. Med..

[7] Schutt-Aine J.C. (1980). Hypothyroid myxedema and hyponatremia in an eight-year-old child: A case report. J. Natl. Med. Assoc..

[8] Wartofsky L. (2006). Myxedema coma. Endocrinol. Metab. Clin. N. Am..

[9] Sun G.E., Pantalone K.M., Hatipoglu B. (2012). Hypothyroidism as a cause of hyponatremia: Fact or fiction?. Endocr. Pract..

[10] Croal B.L., Blake A.M., Johnston J., Glen A.C., O’Reilly D.S. (1997). Absence of relation between hyponatraemia and hypothyroidism. Lancet.

[11] Warner M.H., Holding S., Kilpatrick E.S. (2006). The effect of newly diagnosed hypothyroidism on serum sodium concentrations: A retrospective study. Clin. Endocrinol. (Oxf.).

[12] Schwarz C., Leichtle A.B., Arampatzis S., Fiedler G.M., Zimmermann H., Exadaktylos A.K., Lindner G. (2012). Thyroid function and serum electrolytes: Does an association really exist?. Swis. Med. Wkly..

[13] Asami T., Uchiyama M. (2004). Sodium handling in congenitally hypothyroid neonates. Acta Paediatr..

[14] Upadhyay A., Jaber B.L., Madias N.E. (2006). Incidence and prevalence of hyponatremia. Am. J. Med..

[15] Klein J., Kern W., Fehm H.L. (2001). Fatigue and hyponatremia in a 75-year-old woman: Unusual presentation of hypophysitis. Exp. Clin. Endocrinol. Diabetes.

[16] Shakir M.K., Krook L.S., Schraml F.V., Hays J.H., Clyde P.W. (2008). Symptomatic hyponatremia in association with a low-iodine diet and levothyroxine withdrawal prior to I131 in patients with metastatic thyroid carcinoma. Thyroid.

[17] Krishnamurthy V.R., McDougall I.R. (2007). Severe hyponatremia: A danger of low-iodine diet. Thyroid.

[18] Al Nozha O.M., Vautour L., How J. (2011). Life-threatening hyponatremia following a low-iodine diet: A case report and review of all reported cases. Endocr. Pract..

[19] Nozu T., Yoshida Y., Ohira M., Okumura T. (2011). Severe hyponatremia in association with I(131) therapy in a patient with metastatic thyroid cancer. Intern. Med..

[20] Jo H.J., Kim Y.H., Shin D.H., Kim M.J., Lee S.J., Jeon D.O., Im S.G., Jang S.K., Choi J.Y. (2014). Hyponatremia after thyroid hormone withdrawal in a patient with papillary thyroid carcinoma. Endocrinol. Metab. (Seoul).

[21] Rachoin J.S., Cerceo E.A. (2011). Four nephrology myths debunked. J. Hosp. Med..

[22] Kilpatrick E.S. (2006). Disorders of sodium balance: Hypothyroidism and hyponatraemia: An old wives’ tale?. BMJ.

[23] Verbalis J.G., Goldsmith S.R., Greenberg A., Korzelius C., Schrier R.W., Sterns R.H., Thompson C.J. (2013). Diagnosis, evaluation, and treatment of hyponatremia: Expert panel recommendations. Am. J. Med..

